# Cystic Evacuation and Irrigation

**Published:** 1894-05

**Authors:** 


Cystic Evacuation and Irrigation. By W. Storer How, D. D. S., Philadelphia, Pa. Reprinted from *University Medical Magazine*, March, 1894.

This pamphlet of three pages describes a clever device invented by Dr. How to irrigate the urinary bladder. It consists of a glass reservoir provided with a metal ring and hook for hanging on the back of a chair, and a rubber tube provided with a pinch-cock and terminating in a catheter for introduction into the urethra. The apparatus is simple, portable, compact, and is without valve or ledge for lodgment of stuff. The movement of the fluid is plainly visible, the hydraulic pressure gently variable, and cannot be made excessive in force or volume. Withal it is quite inexpensive.

